# IMPLICATIONS OF TIMING, COMMUNITY, AND DIFFUSION FOR IMMIGRANT HEALTHCARE AND TELEHEALTH EXPERIENCES

**DOI:** 10.1093/geroni/igae098.4388

**Published:** 2024-12-31

**Authors:** Sungjae Hong, Shannon Mejia

**Affiliations:** University of Illinois at Urbana-Champaign, Champaign, Illinois, United States; University of Illinois Urbana-Champaign, Urbana, Illinois, United States

## Abstract

Inequality in access to healthcare among immigrant older adults can evolve into health disparities, resulting in social burdens on society. This study integrates life course and diffusion of innovations perspectives to examine how the timing of immigration, global events, and interdependence intersect with social systems and communication channels to influence healthcare and telehealth adoption. We interviewed 22 Korean immigrant older adults who immigrated to the Chicago Metropolitan Area. The interviews detailed respondents’ life trajectories, including immigration timing, communication channels, and social relationships. Participants also reported perceptions of and experiences with healthcare and telehealth. To discover how perceptions of and experiences with telehealth varied according to life experiences, we conducted the direct content analysis based on the diffusion of innovations and the life course theories. We coded interviews with predefined codes based on the theories but added new codes if needed. Four main themes emerged: 1) Racial/ethnic communities served as the primary communication channel for healthcare information, 2) The difference between the first and 1.5 generations in the trajectories of healthcare service experience, 3) Adult children as the primary driver of telehealth adoption, and 4) The limited scope of authority innovation diffusion, where telehealth services were adopted within the COVID-19 context, but exchanged for in-person healthcare services due to cultural norms. Our findings provide new insight into the lifelong mechanisms of inequalities in healthcare and telehealth and highlights importance of communication channels and community connections in efforts to improve access to and use of telehealth services in immigrant older adult populations.

